# The effect of a natural vaginal product based on honey on the success of intrauterine insemination (IUI) in infertility treatment 

**Published:** 2019

**Authors:** Maryam Kavousi, Nayereh Khadem Ghaebi, Mona Najaf Najafi, Roshanak Mokaberinejad, Zohre Feyzabadi, Roshanak Salari

**Affiliations:** 1 *Department of Persian Medicine, School of Persian and Complementary Medicine, Mashhad University of Medical Sciences, Mashhad, Iran.*; 2 *Department of Obstetrics and Gynecology, Faculty of Medicine, Mashhad University of Medical Sciences, Mashhad, Iran.*; 3 *Clinical Research Unit, Mashhad University of medical sciences, Mashhad, Iran.*; 4 *Department of Traditional Medicine, School of Traditional Medicine, Shahid Beheshti University of Medical Sciences, Tehran, Iran.*; 5 *Department of pharmaceutical sciences in Persian Medicine, School of Persian and Complementary Medicine, Mashhad University of Medical Sciences, Mashhad, Iran.*

**Keywords:** Infertility, Honey, Myristica fragrans, Mace, Intrauterine Insemination (IUI)

## Abstract

**Objective:**

Due to high prevalence of infertility and increasing tendency towards complementary medicine, this study was conducted to investigate the effect of a vaginal natural product based on honey and 1% extract of *Myristica fragrans* on the extent of success of intrauterine insemination (IUI).

**Materials and Methods:**

This non-randomized clinical trial study with a historic control group, was performed on infertile women. In this trial, 159 patients were assigned to the intervention group, and 288 patients were recruited to the control group. All the participants received clomiphene or letrozole from the third up to seventh day of menstruation, and on days 6, 7, and 8, they received human menopausal gonadotrophin (HMG) injections. IUI was performed 36 hours after human chorionic gonadotrophin (HCG) injection. In the intervention group, a natural vaginal product was used besides the above treatments, from menstruation day 7 until the day before performing IUI. Sixteen days after IUI, serum beta HCG was measured to investigate chemical pregnancy, and six weeks following IUI, vaginal sonography was performed to investigate clinical pregnancy.

**Results:**

Analysis showed that the pregnancy rate was higher in the group that receiving the natural product compared to the control group. Chemical pregnancy rate was 18.1% vs. 15.4%, and clinical pregnancy rate was 15.2% vs. 13.8% for intervention and control groups, respectively; but, this difference was not significant.

**Conclusion:**

It seems that the use of this vaginal product for a longer period of time and across several menses cycles before IUI, may produce more positive results. Further studies, however, are needed to be done.

## Introduction

Intrauterine insemination (IUI) is one of the oldest methods of treating infertility. Although use of healthcare services has been increased for treating infertility, in recent years, the prevalence of infertility has not been decreased (Abdelkader and Yeh, 2009 ▶). Around 10-15% of couples around the world suffer from infertility. In Iran, this prevalence is higher than the global average as it was reported to be 17.3-20.2% (Akhondi et al., 2013 ▶; Kazemijaliseh et al., 2015 ▶). The success rate of IUI with homolog sperm varies (10-20% per every cycle) across different studies (Abdelkader and Yeh, 2009 ▶; Monraisin, et al., 2016 ▶; Allahbadia, 2017 ▶; Irani et al., 2018 ▶). IUI is used for treating infertility with different causes including cervical causes, anovulation, endometriosis, immunological causes, male infertility, and unknown factors (Allahbadia, 2017 ▶; Cabry et al., 2017 ▶). Indeed, IUI is considered an intermediate, easier, and cheaper approach in comparison with other methods used for treating infertility such as *in vitro* fertilization (IVF) or intra-cytoplasmic sperm injection (ICSI) (Veltman et al., 2016 ▶). Therefore, more studies should be conducted to enhance the success of IUI.

 Research on traditional treatments that were prescribed from thousand years ago by famous physicians such as Ibn Sina, seems to be essential considering the tendency of patients to alternative medicine. Honey and Mace are among the compounds which are supported by Persian medicine in treating infertility and were proven to be effective in both human and animal studies (Abdelhafiz and Muhamad, 2008 ▶; Zaid et al., 2010 ▶; Zaid et al., 2014 ▶, Lavaf et al., 2017 ▶).

Honey is a sugary supersaturated acidic solution with a high osmotic pressure, which is not suitable for the growth of bacteria and fungi. Indeed, this natural acidity prevents the growth of many pathogenic agents. Honey contains different types of proteins, vitamins, minerals, flavonoids, and alkaloids (Grembecka and Szefer, 2013 ▶; Ranjbaret al., 2015 ▶). In a clinical trial, prescription of a vaginal compound containing honey and royal jelly, alongside normal saline was very effective in treating infertility due to asthenozoospermia (Abdelhafiz and Muhamad, 2008 ▶). Further, adding honey (10%) to cryoprotective solution resulted in enhanced sperm quality after freezing (Fakhrildin and Alsaadi, 2014 ▶; El-Sheshtawy et al., 2016 ▶). In another clinical trial conducted on the effect of vaginal honey on *Candida* vaginitis, it was concluded that vaginal use of honey, while having antibacterial and antifungal effects could maintain and strengthen the normal vaginal flora by increasing lactobacilli (Seifi et al., 2016 ▶). Recent studies about vaginal microbiome reported interference of bacterial species in infertility through next-generation sequencing (NGS) (Campisciano et al., 2017 ▶). Further, pathological changes in the microbiota of the uterus can have a negative effect on the process of implantation, and affect IVF results (Moreno et al., 2016 ▶). Therefore, honey can have a positive effect on fertility by affecting the microbiota of the vagina and in turn the microbiota of the uterus. In a study done by Zaid et al, honey was effective in improving estrogen cycles in mice with artificial ovarian toxicity (Zaid et al., 2014 ▶). In addition, prescription of honey for two weeks resulted in increased epithelium thickness of the vagina and increased weight of the uterus in female mice with ovariectomy (Zaid et al., 2010 ▶).

The external cortex of nutmeg (Myristica fragrans) is called Mace (USDA, 2007 ▶), which contains different types of phytoestrogens, flavonoids and minerals (Zhang et al., 2012 ▶; Chiu et al., 2016 ▶). It has aphrodisiac effects and also antibacterial, antifungal, hepatoprotective, anti-inflammatory, and immunomodulating activities (Tajuddin et al., 2003 ▶; Tajuddin et al., 2005 ▶; Checker et al., 2008 ▶; Zhang et al., 2012 ▶). 

Studies showed that the blood levels of calcium and magnesium diminish during stimulation of ovulation and elevation of estrogen level in infertile women (Grossi et al., 2017 ▶). Further, in different studies, addition of phytoestrogen alongside induction of ovulation with clomiphene could improve the process of ovulation and rate of pregnancy (Shahin et al., 2008 ▶; Shahin and Mohammed, 2014 ▶). In the study done by Tajuddin et al., *M. fragrans* caused a significant increase in sexual activity in male mice (Tajuddin et al., 2003 ▶; Tajuddin et al., 2005 ▶). It seems that this vaginal compound can enhance the chance of fertility by increasing the number of intercourses, in addition to its phytoestrogen properties, which can be effective in ovulation induction. 

In Persian medicine, many infertility drugs are used vaginally, and recent studies proved the effect of vaginal honey on vaginal mucous, vaginal microbiota, uterus microbiota, facilitation of motility and progression of sperm in the vagina (Abdelhafiz and Muhamad, 2008 ▶). Since *M. fragrans* contains a variety of phytoestrogens which can be effective in fertility process. Since a combination of honey and *M. fragrans* is used in Persian medicine as an adjuvant to fertility treatment (Azamkhan, 2008 ▶), this study was conducted to investigate the effect of vaginal natural product made of honey and 1% extract of *M. fragrans* (Mace) on the IUI success rate.

## Materials and Methods

**Study Design**

This clinical trial study which had a historical control group, was conducted from 2016 to 2018 on infertile women candidate for IUI referring to Milad infertility center affiliated to Mashhad University of Medical Sciences, Mashhad, Iran. In the intervention group, the sampling was sequentially performed on patients who referred to the Milad infertility center from 2017/9/23 to 2018/7/1 to perform IUI, and met the inclusion criteria. In the control group, regardless of their pregnancy outcome, the samples were sequentially collected from the archive of this center from patients who referred from 2016/9/22 to 2017/9/22 and met the inclusion criteria. Then, patients’ information was recorded. Patients were matched concerning age and the duration of infertility between the intervention and control groups. This study was approved by the Ethics Committee of Mashhad University of Medical Sciences (IR.MUMS.REC.1395.487), and registered in the center for registering, Iranian clinical trials (IRCT2016111130827N1). In the intervention group, first, the study was explained to the patients, and then, the patients were requested to sign the consent form (if they desired to participate in the study).

**Participants**

The patients were first examined and evaluated by a Gynecologist, and referred to the researcher in Milad Infertility Center. The inclusion criteria included willingness to participate, age <40 years old, infertility duration < 15 years, infertility due to anovulation, infertility due to male factor, and unexplained infertility, sperm count according to total motility function of sperm (TMFS) above 5 million per ml following sperm preparation, normality of hormonal tests, normality of uterine in hysterosalpingography and having at least one open fallopian tube.

The exclusion criteria included body mass index > 35, improper follicle size to inject HCG on day 12 up to 14, and endometriosis. 

**The IUI procedure**

The IUI protocol that was performed for both groups as follows: From day 3 up to 7 of menstruation, they received clomiphene (100 mg/day) or (letrozole 5mg/day) for five days, and on days 6, 7, and 8, they received HMG. The dose of HMG was adjusted based on the ovarian monitoring. On the 9^th^ day, vaginal sonography was performed (if the follicle diameter was below 12 mm, then they received HMG for a second time), and in case follicle ≥ 18 mm on day 12 up to 14 of the cycle, 5000 units of HCG were injected. Then, 36 hr later, IUI was performed with patient's wife sperm sample that was washed and prepared on the same day. To support luteal phase, progesterone (8% vaginal gel one prefilled applicator once daily) was prescribed after IUI.

**Intervention**

In the intervention group, from the 7^th^ day of menstruation until the day before performing IUI, a natural product made of honey and 1% extract of *M. fragrans* (Mace) was applied vaginally using an applicator once per night by the patient herself. A list of the possible side effects of the product was also given to patients. The control group was not treated with the vaginal natural product.

**Randomization and blinding**

Since, the control group was chosen from the archive of the infertility center; This trial was neither a randomized nor a blinded study.

**Drug preparation**

**Plant collection and extraction**

Mace is the red fleshy cup around a nutmeg seed. The plant was verified by the botanist of the Department of Botany Research Center for Plant Sciences in Ferdowsi University of Mashhad, Iran and Mace was prepared from Sobhan Herb center in Mashhad, Iran. High quality honey was prepared from Mashhad, Khorasan Razavi, Iran. Once Mace was prepared, it was crushed and the aqueous extract was extracted through maceration in water. The obtained liquid, extract was pulverized by a freeze dryer. The pulverized extract was then kept at 2-4°C. All of the extraction stages were performed by the research Center for food sciences and industry of Mashhad. 

The amount of plant extract was 2% by weight of the plant.

**Preparation of herbal vaginal product**

Mace and honey were mixed together at 1% (i.e. each 100 g of honey was mixed with 1 g of Mace), and 80-g tubes were filled with the mixture. These stages were performed by a pharmacist at Persian medicine faculty of Mashhad University of Medical Sciences, Mashhad, Iran. Each tube contained 79.2 g of honey and 0.8 g of Mace extract. Once filled, the tubes were given to the patients along with an applicator for vaginal use. The dose of this compound was determined as one applicator per night starting from the 7^th^ day of menstruation and ending on the day before performing IUI. Each applicator which contained 8 g of this product had at least 0.08 g of Mace and 7.92 g of honey.

**Standardizing Mace aqueous extract**

The aqueous extract of Mace was standardized based on total phenol, using Folin-Ciocalteu method (Hosseini et al., 2017). A total of 20 μL of the extract (10 mg/ml), and gallic acid (0, 50, 100, 150, 250, and 500 mg/L) used as the standard, were mixed with 100 μL of Folin-Ciacalteu reagent and 300 μL of 1 M sodium carbonate solution. The final volume was reached 2 ml using deionized water, and after 2 hr, optical absorption of the samples was measured by spectrophotometer at 765 nm. Then, standard curve was drawn for gallic acid and the level of phenolic compounds in the extract was expressed based on mg of gallic acid. The total phenol content in Mace extract was equal to 70.4 mg of gallic acid per gram of the pure Mace extract and the total phenol content in natural product was equal to 0.704 mg of gallic acid per gram based on Maces extract content of the product.

**Microbial tests**

The microbial tests were performed for the product containing honey and Mace in microbiology laboratory of Ghaem Hospital in Mashhad based on the standard method of USP32-NF27 United States pharmacopeia (Pharmacopeia, 2004 ▶). The product was negative for *Escherichia coli*, *Candida albicans*, *Staphylococcus aureus*, aerobic mesophilic bacteria, mold, klebsiella, and yeast.

**Measurements and outcomes**

Patients’ complete history was recorded upon their arrival and physical examination was performed by the researcher. The demographic and basic characteristics of the patients including age of the patient, age of spouse, BMI, duration of infertility, time of IUI, and type of infertility in terms of being primary or secondary, were recorded. Diagnostic tests to measure FSH and LH levels were performed for all patients on the third day of menses cycle. In addition, vaginal sonography was performed on the third and 9^th^ days of menses cycle by a gynecologist, where the number of follicles and the size of largest follicle were recorded. Further, semen analysis test was also performed for the spouse of patients, who were considered normal, if they had the following characteristics, according to the guidelines of the World Health Organization. Sperm volume ≥1.5mL, sperm count ≥15 million /mL, sperm motility (percentage of progressive motility) ≥32% sperm morphology according to strict criteria (Tygerberg) ≥4%.

After recording the history, and performing physical examination, biochemical tests, and sonography, the cause of infertility (being female or male-factor or both or unexplained infertility) was specified and recorded.

The primary outcome included chemical and clinical pregnancy rate. Sixteen days after performing IUI, serum βHCG (human chorionic gonadotropin) was measured using ELISA (enzyme-linked immunosorbent assay) method. Chemical pregnancy was confirmed if βHCG was ≥25 IU/l. Further, six weeks following IUI, vaginal sonography was performed by the gynecologist in the infertility center, where in the presence of pregnancy sac, clinical pregnancy was confirmed. The secondary outcome was the product’s side effects.

The questionnaire of temperament was completed for all patients in the intervention group. The questionnaire for determining the temperament (Mojahedi Mizaj Questionnaire, MMQ) is a standard questionnaire validated in Iran in 2014, and its reliability and validity have been confirmed. The Cronbach alpha coefficient of this questionnaire is 0.71. This questionnaire contains 10 items scoring from 1 to 3. After summing up the scores, a score ≤14 is considered cold temperament, 15 - 18 represents moderate temperament, and ≥19 is considered warm temperament (Mojahedi et al., 2014 ▶).

**Sample size**

Since the success rate of IUI in Milad Infertility Center was about 10%, and bearing in mind that the above-mentioned intervention can enhance the success rate by 10% causing a success rate of 20% (α=0.05 and β=0.2), the sample size in the intervention group and control group was determined as 144 and 288, respectively. Taking 10% loss into account, 159 subjects were considered for the intervention group. Because of limitations of access to drug (natural vaginal product) due to its high cost and the number of patients and in order to stabilize the power of the study, the control group was considered twice the intervention group.

**Statistical analyses**

The recorded data were analyzed by SPSS (Version 16) software. Descriptive statistical methods including central indices, distribution, and frequency distribution were used to present data. Independent t-test was used to compare quantitative variables between the two groups, if data distribution was normal; otherwise, Mann-Whitney test was utilized. To compare the qualitative variables between the two groups, chi-square test, and if required Fischer exact test were used. Logistic regression model was employed to adjust the confounding variables. In all calculations, a p<0.05 was considered statistically significant**.**

## Results

**Figure 1:**
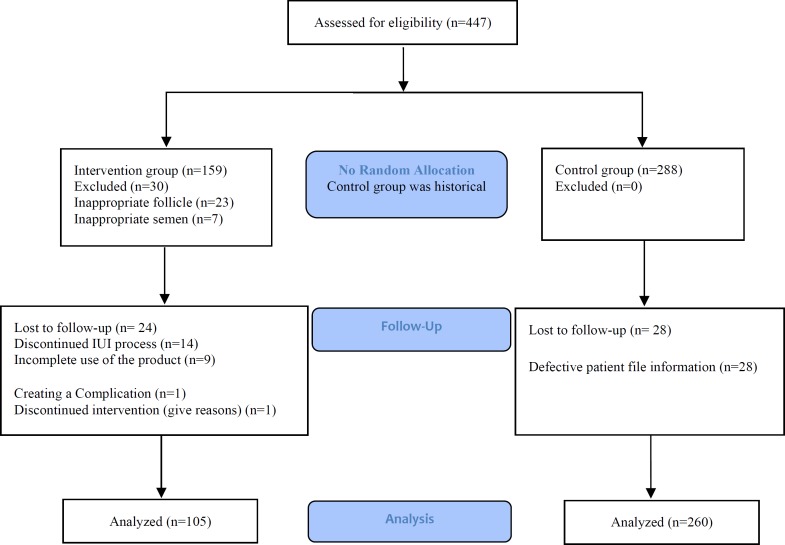
Flow diagram of the study

In this trial, 159 subjects were included in the intervention group. Fifty-four subjects out of 159, were excluded for different reasons (23 subjects due to unsuitable follicle, 7 due to unsuitable sperms, and 14 due to unwillingness to continue to IUI stages, 9 subjects due to not using the product, and 1 subject due to incidence of vaginal pruritus and burning). Finally, 105 individuals were analyzed as the intervention group. In the control group, the information of 288 patients was collected and investigated. Form the control group, 28 patients were eliminated due to incomplete information, and 260 individuals were analyzed as the control group (Figure 1).

The mean age of the patients in the intervention and control groups was 28.3±4 and 28.6±5 years old, respectively, and there was no significant difference between the two groups (p=0.619). 

The mean duration of infertility in the intervention and control groups was 5.36±3 and 4.7±3 years, respectively, and there was no significant difference between the two groups (Table 1).

 There was no evidence of statistically significant differences between the two groups (intervention and control) in terms of chemical pregnancy (OR=1.048, CI=0.538-2.04, P=0.890) and clinical pregnancy (OR=1.120, CI=0.561-2.23, P=0.748) after adjustment of the confounding variables such as the number of follicles and the types of drugs used to stimulate ovulation (letrozole or clomiphone). Other demographic and basic variables are presented in Table1.

**Table 1:** Basic characteristics of patients

	Intervention	Control	P value
Age of patient (years)^a^	28.3±4	28.56±4.8	0.619
Age of husband (years)^a^	32.20±5.3	32.28±5	0.875
BMI (kg/m2)^a^	25±3.5	25.6±4	0.139
Duration of infertility (years)^a^	5.36	4.7±3	0.052
Time of IUI^b^			
First Second Third Fourth	45 (42.9%) 36 (34.3%) 15 (14.3%) 9 (8.5%)	114 (43.8%) 92 (35.4%) 46 (17.7%) 8 (3.1%)	0.627
Kind of infertility^c^			
Primary Secondary	77 (73.3%) 28 (26.7%)	200 (77.2%) 60 (22.8%)	0.431
cause of infertility^c^			
Female factor Male factor Both (female &male) Unknown	29 (27.6%) 29 (27.6%) 11 (10.5%) 36 (34.3%)	59 (22.8%) 71 (27.4%) 19 (7.3%) 110 (42.5%)	0.413
FSH(miu/m)^d^	6.2 (2-19)	6.37 (0.01-28.2)	0.417
LH(miu/m)^d^	4.8 (0.1-26)	4.9 (1.1-26.8)	0.562
Semen analysis^c^, ^e^			
Volume Count Motility Morphology	100 (95.2%) 105 (100%) 88 (83.8%) 101 (96.2%)	247 (95.4%) 258 (99.6%) 208 (80%) 233 (89.6%)	0.958 0.524 0.400 0.060
Follicle count^d^	4 (1-14)	5 (1-15)	<0.01
Maximum size of follicle(mm)^d^	18 (15-26)	18 (15-25)	0.507
Endometrial thickness (mm)^a^	6.9±1.5	7.2±1.6	0.222
Drugs^c^			
letrozole clomiphene	60 (57.1%) 45 (42.8%)	102 (39.2%) 158 (60.8%)	0.003

a Data are shown as mean (SD) analyzed by T tests.

b Data are shown as number (%) analyzed by Mann–Whitney tests.

c Data are shown as number (%) analyzed by Chi-Square Tests.

d Data are shown as median (min–max) analyzed by Mann–Whitney test.

e Comparison of normal values of sperm analysis in intervention and control groups.

**Figure 2:**
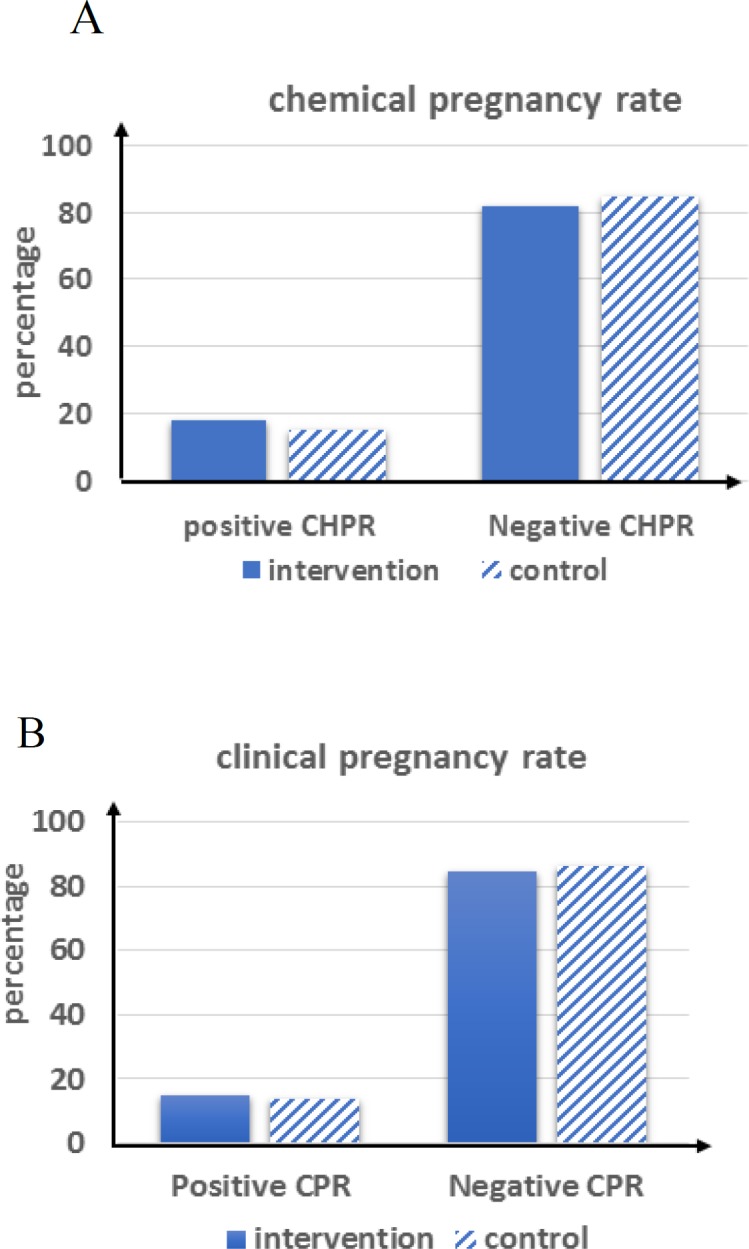
Outcomes in the intervention and control groups (A: Chemical pregnancy rate B: Clinical pregnancy rate).

Chemical pregnancy rate (CHPR) in the intervention and control groups was 18.1% and 15.4%, respectively, with no significant difference between them; however, the level of chemical pregnancy was greater in the intervention group (p=0.524). Clinical pregnancy rate (CPR) was 15.2% in the intervention group and 13.8% in the control group, which were not statistically different; however, CPR was greater (p=0.731) in the intervention group (Figure 2).

CHPR: Chemical pregnancy rate; and CPR: Clinical pregnancy rate.

Although the frequency of different causes of infertility was similar in the intervention and control groups, in the intervention group most of the subjects who became pregnant (both chemically and clinically) had infertility with female factor. On the other hand, in the control group, most pregnancies (chemical and clinical) were due to unexplained causes (Table 2). In the intervention group, patients were compared in general temperament in the positive and negative chemical pregnancy groups; most subjects with positive chemical pregnancy showed a warm temperament, but there was no significant difference between positive and negative chemical pregnancy groups p=0.306). 

Further, in the intervention group, the positive and negative clinical pregnancy groups were compared in general temperament; those with positive clinical pregnancy were warm-tempered, but there was no significant difference between the positive and negative clinical pregnancy groups in general temperament (p=0.144) (Table 3).

**Table 2:** Comparison of the success rate of chemical and clinical pregnancy due to different causes of infertility in the intervention group

		Cause of infertility				
Group		Female factor	Male factor	Both (female &male)	Unknown	P*
CHPR	Positive N (%)	7 (36.8)	3 (15.8)	3 (14.8)	6 (31.6)	0.464
	Negative N (%)	22 (25.6)	26 (30.2)	8 (9.3)	30 (34.9)	
CPR	Positive N (%)	7 (43.8)	3 (18.8)	3 (18.8)	3 (18.8)	0.505
	Negative N (%)	22 (24.7)	26 (29.2)	8 (9)	33 (37.1)	

CHPR: Chemical pregnancy rate; CPR: Clinical pregnancy rate

* Data are shown as number & percent analyzed by Chi-Square Tests.

**Table 3:** Comparison of general temperament (mizajaam) between the two groups positive & negative pregnancy rate in intervention group

Group		Warm	Cold	Temperate	P*
CHPR	Positive N (%)	8 (42.1)	5 (26.3)	6 (31.6)	0.306
	Negative N (%)	20 (24.1)	23 (27.7)	40 (48.2)	
CPR	Positive N (%)	7 (43.8)	3 (18.8)	6 (37.5)	0.144
	Negative N (%)	21 (24.4)	25 (29.1)	40 (46.5)	

CHPR: Chemical pregnancy rate; CPR: Clinical pregnancy rate

* Data are shown as abundance (%) analyzed by Mann–Whitney tests.

In the intervention group, general temperament was compared between the positive and negative chemical pregnancy groups, where most subjects with positive chemical pregnancy showed a warm temperament, but there was no significant difference between positive and negative chemical pregnancy groups (p=0.306). Further, in the intervention group, the positive and negative clinical pregnancy groups were compared in general temperament, where those with a positive clinical pregnancy were warm-tempered, but there was no significant difference between the positive and negative clinical pregnancy groups in general temperament (p=0.144) (Table 3).

Very limited side effects were observed; itching and burning was reported by a patient who had a history of consuming large amounts of spicy seasonings, who was eliminated from the intervention group.

## Discussion

The present study is the first research which evaluated the effectiveness of a natural vaginal product containing Mace and honey in IUI candidate patients. Effect of Mace, as a phytoestrogenic compound alongside honey vaginally coupled with induction of ovulation, was not evaluated before in infertile patients.

The results indicated that the rates of chemical and clinical pregnancy in the group receiving the vaginal product were greater compared to the control group (18.1 vs. 15.4% for chemical pregnancy, and 15.2 vs. 13.8% for clinical pregnancy, respectively), but this difference was not statistically significant. This increase in the success rate of IUI occurred after seven days of using this vaginal product. Possibly, use of this vaginal product in several sequential cycles can produce positive results. Because in the study done by Abdelhafiz et al. using vaginal products containing honey, royal jelly, and bee bread for 2 weeks and in three consecutive cycles, the pregnancy rate was significantly increased compared to the control group (Abdelhafiz and Muhamad, 2008 ▶). Since the study done by Abdelhafiz et al. was the only clinical trial similar to ours, it seems that the short duration of treatment (one week vs. two weeks) and not persisting the treatment (one cycle vs. three cycles) were among the reasons due to which success rate of drug diminished in this study.

In our study, in the control group, the number of patients who received clomiphene was higher than letrozole, which was significantly different from the intervention group. In various studies, the effect of these two drugs on fertility was examined (Roque et al., 2015, Gunn and Bates, 2016). It seems that the effect of clomiphene and letrozole on clinical outcomes and fertility rate in women with unexplained infertility and *polycystic ovary* syndrome* (PCOS)* is equal (Ghahiri et al. 2016 ▶, Eskew et al. 2019 ▶). In our study the chance of chemical pregnancy and clinical pregnancy in the intervention and control groups after adjusting for the confounding variables (i.e. follicle count and types of drugs) used for stimulate ovulation (letrozole or clomiphone)) did not have any significant difference.

Various studies suggested that cervical infection caused by gram-negative bacteria can have a negative effect on assisted-reproductive techniques (Salim et al., 2002 ▶; Moreno and Franasiak, 2017 ▶). Moreover, removal of cervical mucus before IUI, was shown to increase the rate of fertility twice with the possible mechanism of removing the microbes that contaminate the catheter tip which causes impaired implantation. However, the presence of cervical mucus prevents sperms to return to vagina following insemination. In this way, it increases the level of sperm present inside the fallopian tube. Therefore, through using honey vaginally, a positive effect on fertility can be achieved without physical removal of vaginal mucus by improving the microbiota. Although different studies indicated that honey improves the uterus microbiota in (Brudzynski and Sjaarda, 2015 ▶; Seifi et al., 2016 ▶), long-term consumption of this product can possibly increase the success of infertility treatment because in the study done by Abdelhafiz, et al., use of a vaginal product containing honey for 2 weeks had positive results (Abdelhafiz and Muhamad, 2008 ▶).

In the intervention group, 43.7% of those who became pregnant had female factor infertility, but in the control group this figure was 24.7%. Although this difference was not statistically significant, it seems that the vaginal product can have a greater effect on female causes. In other words, vaginal use of this product, by influencing the normal flora of vagina or its microbiota, may lead to successful of IUI in individuals with female factor infertility. On the other hand, the results of the study done by Abdelhafiz et al indicated that the vaginal product containing honey, royal jelly, and flower pollen was effective in infertile patients with male causes by increasing sperm motility in vagina without any IUI (Abdelhafiz and Muhamad, 2008 ▶).

Considering general temperament, most of the individuals who became pregnant in the intervention group had a warm temperament, while those with cold temperament had the minimum number of pregnancy (42 vs 26% for ? and ?, respectively). This result is somehow similar to those reported by Sohrabvand et al; they reported that the most common temperament in their study which was conducted on infertile women referring to an infertility clinic, was cold (Sohrabvand et al., 2014 ▶). Since the temperament of our herbal product is warm, it seems that the short duration of drug use is one of the causes of diminished IUI success in individuals with cold temperament compared to those with warm temperament. Possibly, if the vaginal product was used for a longer time for several consecutive cycles before IUI and lifestyle and food orders were also corrected for the temperament, this product could have a greater effect on IUI success by making the temperament warm.

Investigating the effect of the product on infertility in cases with female, male, and unexplained factors, was the strengths of this study. On the other hand, Abdelafiz et al studied individuals with only male factor infertility, and the effect of drug was not evaluated in cases of other causes of infertility. The weak point of this study were limitation the number of patients that caused in order to stabilize the power of the study. Therefore, in the present study, a historical control group considered twice the intervention group. So, the randomization and blinding were not possible in the current study. The other weak point of this study was short duration of drug application. In addition, the absence of facilities for screening the uterus microbiota before and after the intervention was the other limitation of the study. 

Considering the increase in the success rate of IUI in the intervention group, although non-significant, further studies should be conducted with a longer treatment duration for at least two weeks in each cycle of fertility and for at least three cycles. Also, the oral consumption of this compound for the same time as vaginal application may improve the response to treatment given the oral effect of phytoestrogens on ovulation induction.
